# Harmony Perception in Prelingually Deaf, Juvenile Cochlear Implant Users

**DOI:** 10.3389/fnins.2019.00466

**Published:** 2019-05-08

**Authors:** Victoria Zimmer, Jesko L. Verhey, Michael Ziese, Martin Böckmann-Barthel

**Affiliations:** Department of Experimental Audiology, Otto von Guericke University of Magdeburg, Magdeburg, Germany

**Keywords:** cochlear implants, musical harmony, consonance and dissonance, musical syntax, perception, cadences

## Abstract

Prelingually deaf children listening through cochlear implants (CIs) face severe limitations on their experience of music, since the hearing device degrades relevant details of the acoustic input. An important parameter of music is harmony, which conveys emotional as well as syntactic information. The present study addresses musical harmony in three psychoacoustic experiments in young, prelingually deaf CI listeners and normal-hearing (NH) peers. The discrimination and preference of typical musical chords were studied, as well as cadence sequences conveying musical syntax. The ability to discriminate chords depended on the hearing age of the CI listeners, and was less accurate than for the NH peers. The groups did not differ with respect to the preference of certain chord types. NH listeners were able to categorize cadences, and performance improved with age at testing. In contrast, CI listeners were largely unable to categorize cadences. This dissociation is in accordance with data found in postlingually deafened adults. Consequently, while musical harmony is available to a limited degree to CI listeners, they are unable to use harmony to interpret musical syntax.

## Introduction

For young humans, music represents a beneficial factor in language, social, creative development (see Hallam, 2010), and plays a role in adolescents’ mood regulation (Saarikallio and Erkkilä, 2007). Although cochlear implant (CI) users face substantial degradations of sound details, many of them enjoy listening to music, and its contribution to their quality of life has been reported repeatedly (Leal et al., 2003; Lassaletta et al., 2007). This was mainly studied in adults but a positive attitude toward music may be regarded as an important objective also for young prelingually deaf who acquire their musical experience via the CI only. However, music appreciation is deteriorated by the unavoidable reduction of spectral and dynamical sound information coming with electrical stimulation (for a review, see Limb and Roy, 2014), partly due to technical shortcomings such as the limited number of electrodes and reduced fine temporal details which result in reduced pitch cues, and partly due to neuronal deprivation over the period of deafness. CI listeners perceive pitch less accurately than normal-hearing (NH) listeners (Pretorius and Hanekom, 2008; Kang et al., 2009), as well as other spectral parameters in music, such as melody contour (Galvin et al., 2009) and instrument timbre (Kang et al., 2009; Brockmeier et al., 2011). Roy et al. (2014) found similar results in CI children, who exhibited difficulty in discriminating pitch and timbre, but less so for discriminating chord sequences.

Western music makes use of distinct tone combinations that may convey pleasantness or rest, as opposed to agitation or tension, commonly seen as different degrees of dissonance. Discrimination and preference of two-tone intervals or chords combining three or more tones was only addressed in very few studies involving CI listeners, showing, for example, that they may be able to discriminate chords from natural piano recordings, but with significantly more effort than NH listeners (Brockmeier et al., 2011; Böckmann-Barthel et al., 2013). CI users may also assign valences to these chords (Brockmeier et al., 2011). The Mu.S.I.C. Perception test used in Brockmeier et al. (2011) was replicated in children with comparable results (Stabej et al., 2012), although the authors reported only the average valence of all chords. Roy et al. (2014) investigated five musical discrimination tasks in young CI listeners at an age of about seven and NH peers. Whereas on average CI listeners were outperformed by the NH peers, both groups were on a level in distinguishing three-chord sequences that differed only in the central chord. Whereas chords may be distinguishable through a CI, the perceived harmonic valence remains unclear.

The concept of harmony has been defined more precisely in music literature as the “combining of musical notes, simultaneously, to produce chords, and successively, to produce chord progressions” (Dahlhaus, 1980). Thus, this definition comprises “vertical” consonance of simultaneous musical tones as well as the “horizontal” relation of consecutive tone combinations. Vertical consonance itself consists of sensory factors such as roughness (Plomp and Levelt, 1965), and music-cultural factors acquired implicitly by exposition (Tramo et al., 2001; Cook et al., 2007). With respect to vertical consonance, the major triad, containing a note four semitones and another one seven semitones above the root note, is generally regarded as the most consonant chord. Several studies showed that (i) minor triads are perceived as somewhat less consonant than major triads, and (ii) that augmented and diminished are rather dissonant (Roberts, 1986; Cook et al., 2007; Johnson-Laird et al., 2012), in accordance with music theory.

The “horizontal” succession of tone combinations structures a musical piece, along with the melody, by means of harmonic tension and release. It requests characteristic chord sequences that indicate the conclusion of a musical phrase, and thus carry syntactic information, just as a full-stop in speech (Rockstro et al., 1980). The most general archetype is the authentic (or perfect) cadence, which is concluded by the dominant (a major chord with a root on the fifth step of the scale) followed by the tonic chord (on the root note of the scale). The present study addresses both the consonance of isolated chords and their functional role in authentic cadences. Following Tramo et al. (2001), we restrict the use of the term “consonance” to the vertical impression that can be derived from isolated chords. In contrast, “harmony” also comprises the horizontal arrangement of chords and their functional roles.

Koelsch et al. (2004) addressed the availability of such horizontal harmony to CI users by means of event-related brain potentials (ERP). The presence of components associated with musical syntax suggested that a certain harmonic irregularity, the Neapolitan sixth chord, is indeed transmitted, although the respective ERP amplitudes are considerably smaller than in NH listeners. Knobloch et al. (2018) varied authentic cadences by replacing the final tonic chord by an unexpected, ill-fitting chord. NH listeners easily detect such an alteration. In contrast, the vast majority of CI listeners were unsuccessful in this task, no matter whether the final chord was a vertically consonant transposition of the tonic, or a vertically dissonant chord. This finding indicates a different perception of chords within a cadence in contrast with chords in isolation, since the CI listeners judged the major chords (which ended the original cadences in one experimental condition) as clearly more consonant than the more dissonant types when presented alone.

Difficulties to perceive musical harmony through a CI may also depend on musical experience. In NH listeners, substantial aspects of musical harmony perception develop with age. For example, the identification of the musical modes major and minor with happy and sad emotions, respectively, is, in accompanied melodies, available by the age of eight but not at the age of four (Gregory et al., 1996). Horizontal aspects of harmony are significantly more subject to development. Processing of authentic cadences is not completely available to children at the age of 5 years when compared to children at 11 years (Schellenberg et al., 2005). These authors also concluded that acquisition of knowledge on horizontal aspects of harmony mostly relies on implicit learning. Only sensory consonance of isolated tone combinations is regarded as predominantly innate (Trainor and Heinmiller, 1998, however, see Plantinga and Trehub, 2014).

Such findings suggest that lack of exposure to music contributes to the above mentioned difficulties of CI listeners to gather harmonic syntax (Knobloch et al., 2018). Whereas these data were obtained from experienced, postlingually deafened listeners who were exposed to music prior to implantation, it is widely unclear to what degree the harmonic concepts, such as vertical consonance or horizontal cadences, might be transferred from previous acoustical experience to the perception of the CI signal. The findings of Knobloch et al. (2018) argue against such a benefit, because except for a single case their CI listeners were largely unable to recognize authentic cadences. It is, however, possible that the comparison with the acoustic music experience renders the music experience via the CI uncomfortable and confusing, because the dissimilar sound sensation of the electrical stimulation might conflict with the memory of previously experienced musical nuances. In this case, prelingually deafened CI listeners might respond closer to NH listeners especially with respect to deviant cadences.

In order to separate the contribution of prior musical experience from the signal-driven percept, this study focused on prelingually deafened children, whose only hearing experience is through a CI. This study includes three experiments, each focusing on a different aspect of harmony perception. Isolated chords had to be discriminated in the first experiment, providing a prerequisite for a correct perception of cadences. The hypothesis is that the CI may be able to do this task, although less accurate than the NH listeners, since the representation of the stimuli in the CI should be different for the different chords. The second experiment tested vertical consonance by investigating which chord types were preferred as more pleasant over others. If vertical harmony was preserved by the degraded CI signal, CI listeners with some musical experience, at least implicitly acquired, would actually prefer the same chords as their NH peers. The third experiment investigated the ability of the CI users to evaluate the musical correct chord progression in the form of authentic cadences with respect to horizontal harmony. If previous musical experience interfered with the experience of music through the CI, the prelingually deaf participants would be expected to be more successful here than the postlingually deaf adults in Knobloch et al. (2018). The cohort of NH listeners covered the hearing age of the CI listeners and was included in the study to test if the tasks were appropriate even for the youngest participants.

## GENERAL METHODS

### Participants

Cochlear implant listeners were recruited from regular follow-up visitors at the university hospital in Magdeburg and the Cecilienstift Cochlear Implant Rehabilitation Center in Halberstadt. Twelve children with bilateral congenital or prelingual deafness (four males and eight females) participated in the study. Except for listener CI02, all were implanted bilaterally and used both devices in daily life. Their age ranged between 7.6 and 18.9 years, with a mean of 14.4 ± 3.4 years. They had a CI experience between 6.0 and 17.2 years with a mean of 12.5 ± 3.3 years which is referred to as hearing age below. CI experience was highly correlated with age at testing, *r* = 0.989, *p* < 0.01. Seven of them used devices by MED-EL, four by Cochlear and one by Advanced Bionics. All of them were profoundly deaf by 2 years of age. No cases of known neurologic disorders or meningitis were included. Demographic and device data are specified in Table 1. All CI listeners spoke German as their first language. None of them had received any musical training beyond school, which usually covers some singing and basic musical knowledge. In particular, they did not participate in any individual instrument training.

**Table 1:** Demographic and device data of the CI participants.

Participant	Age at testing (years)	Age at first fitting (years)	Hearing age (years)	Type of CI	CI sound processor	CI sound processing strategy	Active channels *Right/Left*	Lower cut-off frequency in Hz	Upper cut-off frequency in Hz
CI01	15.6	1.6	14.0	Combi40+^a^	OPUS2	FS4	12/12	100	8500
CI02	16.8	2.1	14.7	Combi40+^a^ unilateral	OPUS2	HDCIS	11/-	200	7000
CI03	15.6	1.8	13.8	Combi40+^a^	OPUS2	FS4	12/12	100	8500
CI04	15.6	1.8	13.8	Combi40+^a^	OPUS2	FS4	12/12	100	8500
CI05	13.7	2.0	11.7	PULSAR^a^	OPUS2	FSP	12 / 12	100	8500
CI06	15.9	2.3	13.6	Combi40+^a^	OPUS2	FSP	12/12	100	8500
CI07	18.9	1.8	17.2	C124M^b^	CP810	CIS	12/12	200	7146
CI08	13.5	3.0	10.5	PULSAR 1000^a^	OPUS2	FSP	12/10	100	8000
CI09	17.8	2.3	15.4	C124RE^b^	CP910	ACE	18/18	188	6813
CI10	7.6	1.6	6.0	HiRes90K Helix^c^	Harmony	HiRes-Sw/Fidelity120	16/16	333	6665
CI11	8.5	1.8	6.8	C124RE^b^	CP910	ACE	22 / 22	188	6938
CI12	13.2	1.0	12.2	C124M^b^	CP910	ACE	22/22	188	7938


*The lower and upper cut-off frequencies are the same for both ears in all listeners except CI02 who is implanted unilaterally. Footnotes in column “Type of CI” indicate manufacturers: MED-EL (a), Cochlear (b), Advanced Bionics (c)*.

Twenty-four NH children (14 males, 10 females) without musical training beyond school served as control group. They were recruited through internet announcements. Their age ranged between 5.8 and 18.2 years with a mean of 12.3 ± 3.5 years, thus matching the hearing age of the CI group. Normal hearing was verified prior to the experiments with pure-tone audiometry at audiometric frequencies from 125 to 8000 Hz. To be considered as a NH listener, all thresholds had to be better or equal to 25 dB hearing loss in both ears. All NH children spoke German as their first language. All 12 CI users and 24 NH listeners completed three experiments described below. Written informed consent to the study was obtained before the measurement by a parent or legal guardian or, in the case of the older children, the participant himself. The study was approved by the local institutional review board to fulfill the Declaration of Helsinki.

### Apparatus

The chords used in all three experiments were constructed of four harmonic complex tones, as in our previous study with postlingually deaf adults (Knobloch et al., 2018). Each harmonic tone complex consisted of the fundamental frequency (F0) and the next four partials (2 F0 to 5 F0) with random phases and a decay of 6 dB per partial.

The children were tested separately in a large sound-attenuated room. Sounds were presented through a single frontal monitor loudspeaker (Reveal R5A, Tannoy Ltd., Coatbridge, United Kingdom) at a distance of 1.3 m to the forehead of the child. The sound level was chosen to be clear enough and comfortable to the listener, and did not exceed 85 dB SPL. If the child preferred so, a parent was allowed to be present within the room but outside the child’s view and without the opportunity to interact.

Stimulus presentation and response collection were administered by a MATLAB graphical user interface (The Mathworks Inc., Natick MA, United States). Instructions were provided and responses were given on a touchscreen monitor display in front of the listener.

### Procedure

In order to familiarize the children with the tasks and the setup, the experiment was preceded by a short visual two-interval, two-alternative task that was a visual analog to the first discrimination experiment and used the same graphical user interface. Two pictures (drawn from a cartoon animal set of an orange mouse, a blue elephant, and a yellow duck) were shown in succession and the instruction: “Are the following images identical?” After each presentation, the two answer buttons marked “Ja” (“Yes” in German) with two identical pink triangles and “Nein” (“No” in German) with two different symbols (a pink triangle and a yellow circle) were shown. The symbols were added to enable even children without perfect reading to respond adequately. It was evident after only a few presentations that all children (including the youngest) responded perfectly and were thus able to perform the discrimination task.

Blocks of the three experimental tasks were interspersed. In each experiment, the listener started the next trial by pressing the button marked “Listen.” Stimuli started then without any cue sound after 500 ms. Repeated listening was allowed in all experiments, but this was rarely used by most of the CI and NH listeners. The specific tasks are described in detail in the following sections, and include description of the statistical analysis specific to each experiment.

Statistical analysis were performed with SPSS Statistics version 24 (IBM, Armonk NY, United States). For all experiments Pearson correlations were used to analyze age correlations.

## Experiment 1

### Methods

**FIGURE 1:**
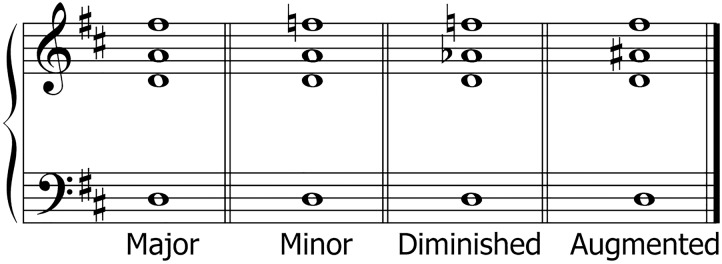
Musical scores for the four different chords used in experiments 1 and 2.

Experiment 1 assessed the discrimination of two chords. All chords were presented in open harmony. At least six semitone steps separated adjacent notes within each chord. Four chord types were used: major, minor, augmented, and diminished chords. Scores are shown in Figure 1. The fundamental F0 of the chord root was randomly chosen from five values separated by one semitone step: 125, 132, 140, 148, and 157 Hz. Each chord had a duration of 1500 ms including 80 ms raised cosine ramps at the beginning and end. The two chords of a pair were separated by silence of 2000 ms. The chord pairs were generated on demand by a MATLAB routine. Equal numbers of all chord types were presented in 48 pairs, 24 comprising identical chords and 24 comprising differing chords. Thus, each chord occurred in three identical pairs and six times in a differing pair. They were separated in four blocks of 12 trials each.

The instruction of the graphical user interface read (English translation of the original German instruction): “You will hear two sounds one after another. Are they the same?” and two answer buttons as above.

For the statistical analysis of the data, each response of type “Yes” (same) following an identical pair was considered as a hit, and each response of type “Yes” following a differing pair as a false alarm. For each participant the occurrence rates of hits (HR) and false alarms (FR) were converted into a sensitivity index according to signal detection theory as *d*′ = z(HR) – z(FR) (Macmillan and Creelman, 1990). In order to avoid infinite values, perfect false alarm rates of 0 were replaced by 1/(2n), n being the number of differing pairs, and perfect hit rates of 1 were replaced by 1 – 1/(2m), m being the number of identical pairs (cf. Stanislaw and Todorov, 1999). With this correction, perfect performance results in a value of *d*′ = 4.07. A one-sample *t*-test was used to examine if *d*′ was different from zero, i.e., chance performance, in the groups. To examine a possible bias in the answer behavior, the decision criterion c = [z(HR) + z(FR)]/2 was also calculated (Macmillan and Creelman, 1990). A listener’s *c* < 0 would indicate a bias toward judging even differing pairs as identical. Again, a one-sample *t*-test examined if c was different from zero in the groups. An independent-samples *t*-test was used to compare the mean *d*′ values of the two groups.

### Results

**FIGURE 2:**
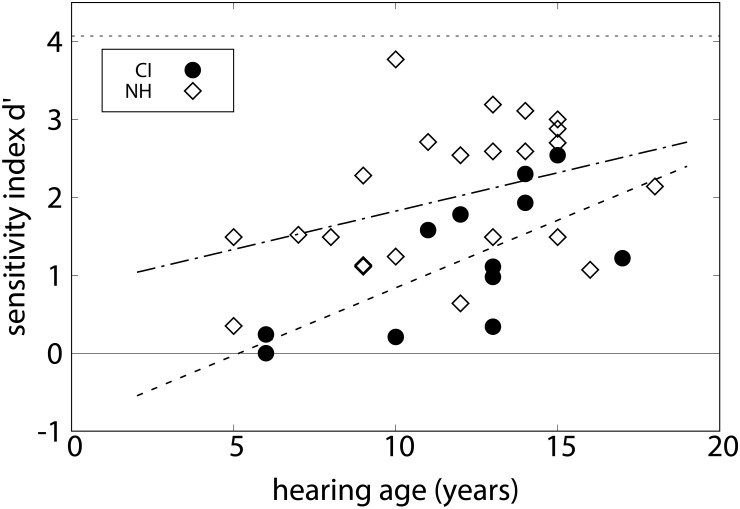
Sensitivity index *d*′ for experiment 1 (chord discrimination task) for individual CI (circles) and NH listeners (diamonds), as a function of hearing age. The lines show linear regressions to the data.

In the discrimination of chord types, five out of twelve CI listeners scored a sensitivity index *d*′ < 1 for the ability to discriminate pairs of single chords. According to signal detection theory, this means that the probability density functions of the responses to targets and distractors are separated by less than one standard deviation (Stanislaw and Todorov, 1999). In other words, these listeners did not discriminate the chord types. In contrast, in the group of 24 NH listeners, only two listeners performed at such a low level. The CI listeners obtained a group mean *d*′ = 1.19 (*SD* = 0.86). The NH control listeners reached a group mean *d*′ = 2.00 (*SD* = 0.90), indicating that they were mostly able to discriminate the different chords. One-sample *t*-tests showed that for both groups the sensitivity indices were significantly above chance level, *t*(11) = 4.77, *p* < 0.01 for the CI listeners and *t*(23) = 10.85, *p* < 0.001 for the NH listeners. The performance of the CI listeners was significantly lower than that of the NH listeners, *t*(34) = 2.58, *p* < 0.05. In order to display the perceived differences of the chord types, Table 2 collects the correct rejection rates of the various differing pairs rated as different. The NH listeners discriminated the pairs involving a minor chord with greater accuracy than the others. The pattern is similar but less pronounced in the CI listeners. Figure 2 shows *d*′ values as a function of hearing age for individual subjects. A significant correlation was found in CI listeners between hearing age and *d*′, *r* = 0.654, *p* < 0.05. The correlation with age at testing was also significant, *r* = 0.654, *p* < 0.05. For the NH control listeners, the correlation between age and *d*′ was not significant, *r* = 0.378, *p* > 0.05. The mean decision criterion testing a tendency toward one of the two alternatives was *c* = -0.25 (*SD* = 0.64) on average for the CI listeners. This value was not significantly different from zero, *t*(11) = -1.33, *p* > 0.05. For the NH listeners, however, the mean decision criterion was *c* = -0.34 (*SD* = 0.47), which was significantly different from 0, *t*(23) = -3.68, *p* < 0.001, indicating a bias toward judging the chords as “same.”

**Table 2:** Detailed percent correct rejection rates, i.e., differing pairs rated as different, of experiment 1, for the combinations of chord types.

	CI			NH		
	Minor	Augmented	Diminished	Minor	Augmented	Diminished
major	73%	65%	63%	90%	44%	63%
minor		63%	75%		88%	90%
augmented			56%			55%


*CI listeners (left) and NH listeners (right)*.

### Discussion

Although CI listeners on average were able to discriminate the chords, discrimination performance was significantly poorer than that of the NH peers. This was expected, since CI listeners typically face difficulties in tasks that rely on accurate spectral information (Limb and Roy, 2014). CI listeners often exhibit pitch difference limens for single tones on the order of several semitones (see, e.g., Pretorius and Hanekom, 2008; Kang et al., 2009). In the present experiment, a given pair of chords differed by only one or two semitones in the top two notes of the chords. Taken this small difference into account, an even larger discrepancy between the two groups might therefore have been expected. In some cases, children listening through a CI have been reported to discriminate chords on the same level as their NH peers (Roy et al., 2014). In their experiment, the target chords were framed by harmonically related major chords. Whereas this framing is not expected to facilitate the discrimination, the good performance might be related to large contrasts between the center chords in the frequency range. The present study showed that when using only chords with the same fundamental, still half of the CI listeners were able to discriminate these chords, although with more difficulty than the NH peers. It should be noted that the NH listeners showed a significant bias toward judging the pairs as same, underlining that even to them the stimuli sounded rather similar.

## Experiment 2

### Methods

Experiment 2 addressed the preference of chord types. To this end, 48 differing pairs of chords were presented in 4 blocks of 12 trials each. The sounds were identical to those used in experiment 1. Each of the four chords was thus presented 24 times. After a pair, the interface display read (original in German) “Which of the sounds sounded more pleasant?” and provided two buttons numbered “1” and “2.” Again, repeated listening was allowed. A preference score was determined for each chord type in each listener by subtracting the number of pairs in which this chord was judged as unpleasant from the number of pairs in which it was judged as pleasant (cf. Tufts et al., 2005). Possible score ranged between -24 and +24. A repeated-measures analysis of variance (ANOVA) with the factor chord type and Greenhouse–Geisser correction tested the null hypothesis that all chord types were preferred equally. It was further hypothesized that the chord preferences were of the following decreasing order: major – minor – diminished – augmented, thus matching the consonance expected from music theory and literature (e.g., Roberts, 1986). Therefore, the score values were ordered accordingly, and a trend line of the scores in this order was constructed for each participant: A steeper negative slope *a* indicates a stronger preference for the expected order of chord types. An independent-samples *t*-test was used to compare the means of the slope *a* of the two groups.

### Results

**FIGURE 3:**
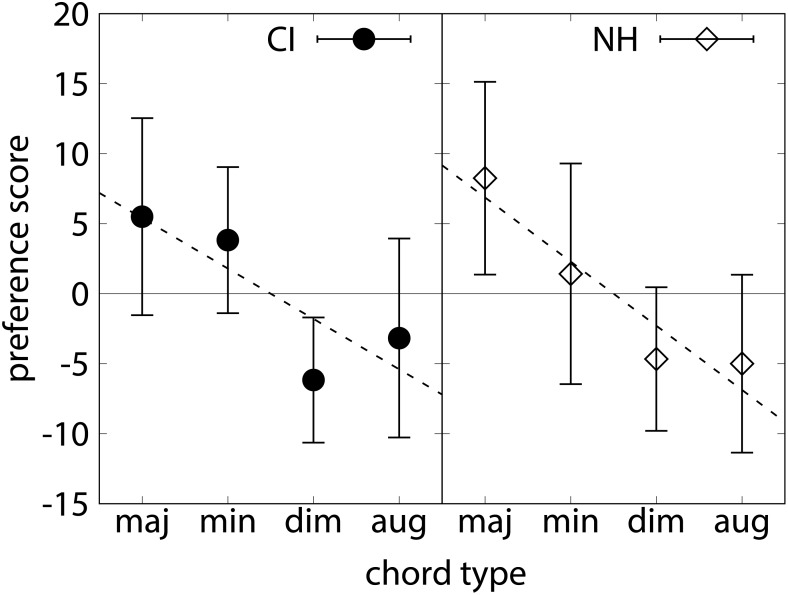
Mean preference scores of the different chord types in experiment 2 (chord preference task), CI listeners (left panel) and NH listeners (right panel). Error bars indicate standard deviation.

**FIGURE 4:**
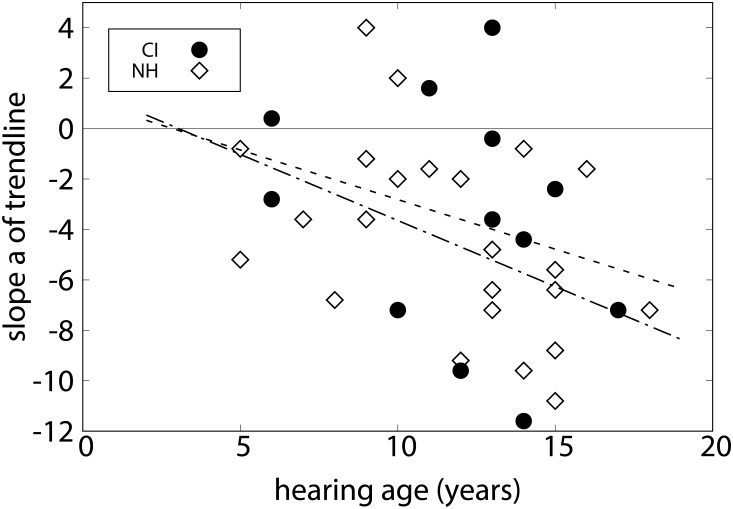
Slopes of the chord preferences of experiment 2 are shown as a function of the hearing age for CI listeners (circles) and NH listeners (diamonds) with corresponding correlation lines. The falling correlation lines indicate a tendency for increasing preference for more consonant chords for more experienced listeners in both groups.

On average, NH listeners preferred the major chord over the minor chord, the diminished chord, and the augmented chord (Figure 3), consistent with the hypothesized order. A repeated-measures ANOVA showed that the ratings of the different chord types depended significantly on the factor chord type, *F*(2.43,55.84) = 15.91, *p* < 0.001. In *post hoc* tests, scores for major and minor chords were significantly higher than those for diminished and augmented chords (*p* < 0.001). The difference of the major and minor chord just failed to reach significance (*p* = 0.051), all other differences were not significant. The CI listeners on average also preferred major and minor chords over augmented and diminished chords, and were thus also consistent with the suggested order. Again, the factor chord type was significant, *F*(1.66,18.26) = 7.57, *p* < 0.01. In *post hoc* tests, scores for the major and minor chords were significantly higher than those for diminished chords (*p* < 0.05). No other differences were significant. Because these *post hoc* tests did not reveal more differences between the chords due to the variability of the data, the slope of preference scores across chords was calculated for individual listeners. The group mean slopes were *a* = -4.58 (*SD* = 3.95) for the NH listeners and *a* = -3.60 (*SD* = 4.67) for the CI listeners, respectively. No significant difference was found between the two groups [*t*(34) = 2.58, *p* > 0.05]. Figure 4 shows slope values as a function of hearing age for individual listeners. For the CI listeners, the slope was not significantly correlated with hearing age, *r* = -0.248, *p* > 0.05, and also not with age at testing, *r* = -0.237, *p* > 0.05. For the NH children, however, a significant correlation between the slope and the age was found, *r* = -0.480, *p* < 0.05.

### Discussion

Previous studies have shown that NH listeners, whether musically trained or not, judge chord types according to the following order of increasing dissonance: major, minor, diminished, and augmented chord (Roberts, 1986; Johnson-Laird et al., 2012). Our data on the NH control listeners were consistent with this order, although the diminished and augmented chords were assigned similarly low preference scores. It was further hypothesized that children listening through a CI showed similar patterns of preference as NH listeners. Indeed, our CI listeners also preferred major and minor chords over diminished and augmented chords. The slope in judgment scores was slightly, (but not significantly) lower in CI than in NH listeners. Knobloch et al. (2018), using similar stimuli but with two additional chord types, reported that adult postlingually deaf CI listeners preferred major chords over all other chord types. In contrast NH adults preferred major and minor chords over other chord types, including diminished and augmented chord types, as expected. The present data were consistent with Knobloch et al. (2018), except that young, prelingually deafened CI listeners assigned higher scores to the minor chord, consistent with the above-mentioned musicological and psychoacoustical expectations.

## Experiment 3

### Methods

**FIGURE 5:**
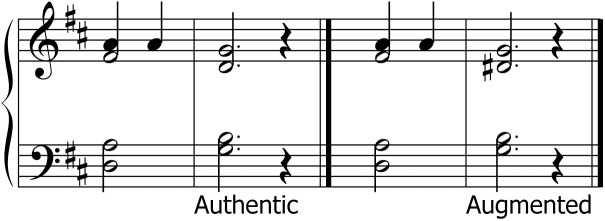
Exemplary score of the two final bars of a song harmonization, containing an authentic V-I cadence and a cadence ending with an augmented chord (right).

Experiment 3 used the final lines of eight different children’s tunes, each of which had a different key between D major and A flat major in semitone steps (see Table 3). The melody of all sequences ended on the root note of the key. Simple four-part harmonisations of all melodies were composed. Apart from the actual key, the last two chords of every harmonization were identical in all songs, as displayed in Figure 5, forming an authentic dominant-tonic cadence. The final tonic chord was in close root position, with four semitone steps separating the lowest two notes. The frequencies of the individual partials ranged from 100 to 2330 Hz. The scores of the examples are provided as Supplementary Material. In 50% of the presentations, these original sequences were presented, ending with the tonic chord. In the other 50% of the presentations, the final chord of the sequence was replaced by either an augmented, or a diminished chord with the same root note, providing a music-syntactically irregular ending. Each of the eight different sequences was presented four times ending in the original version, two times ending with an augmented chord, and two times ending with a diminished chord, resulting in 64 total trials. According to the tune, the duration of each sequence ranged from 7.5 to 8.2 s, and the duration of the final chord ranged from 950 to 1370 ms. An exception was “Sandmann” with the final chord lasting 1800 ms due to the slow tempo and triple metrum of the tune. The durations of the original and altered versions were identical for every tune.

**Table 3:** Keys and corresponding fundamental frequencies of tonic root notes of the eight different children’s tunes used in experiment 3.

Tune	Key	F0 of root note
Guten Abend, gut’ Nacht (Brahms’ Lullaby)	D major	148 Hz
Schneeflöckchen, Weißröckchen	E b major	157 Hz
Spannenlanger Hansel	E major	166 Hz
Sandmann, lieber Sandmann	F major	176 Hz
Fuchs, Du hast die Gans gestohlen	F # major	187 Hz
In der Weihnachtsbäckerei	G major	198 Hz
Happy Birthday	A b major	210 Hz
Grün, grün, grün, sind alle meine Kleider	A major	222 Hz


After each sequence, the interface display was “Did the tune end good or bad?” It was explained to the children that “good” was synonymous with “pleasant,” “satisfying,” or “familiar,” whereas “bad” was synonymous with “unpleasant,” “dissatisfying,” or “unfamiliar.” One of the two answer buttons displayed the word “Good” accompanied by a green, smiling face, and the other “Bad” accompanied by a red, sulking face.

The sequences of Experiment 3 were generated in MIDI format. These MIDI files were resynthesized with the same harmonic tone complexes as above using a MIDI to WAV freeware MATLAB code (Schutte, 2012) and saved as wav files. During the experiment, these wav files were played back.

For the data analysis, each response of “Good ending” following an original sequence was considered as a hit, and each response of “Good ending” following a sequence with an augmented or diminished ending as a false alarm. Otherwise the data analysis is analogous to experiment 1, i.e., for each participant, the occurrence rates of hits and false alarms were converted into a sensitivity index *d*′ and perfect false alarm rates and perfect hit rates were replaced as before to avoid infinite values. With the correction, perfect performance here results in values of *d*′ = 4.31. To examine a possible bias in the answer behavior the decision criterion c was also calculated. A decision criterion *c* < 0 indicated a bias toward judging even the altered versions of the tunes as “good.”

### Results

**FIGURE 6:**
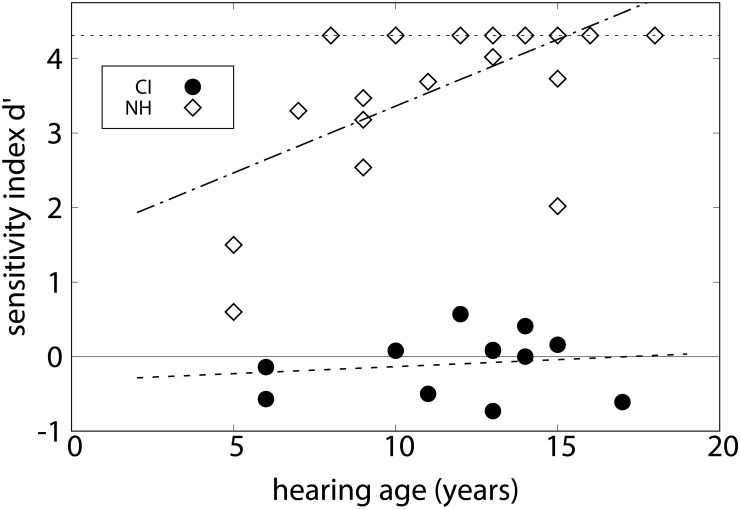
Individual sensitivity indices *d*′ of the cadence judgment task are shown as a function of hearing age for CI listeners (circles) and NH listeners (diamonds) with corresponding correlation lines.

Experiment 3 assessed whether the listeners categorized authentic cadences differently from altered versions. None of the CI listeners performed at *d*′ > 1.0, i.e., none of them discriminated the cadences successfully. The mean *d*′ = -0.10 (*SD* = 0.42) was not different from zero, representing chance level, *t*(11) = -0.80; *p* > 0.05. In contrast, the NH listeners reached a very high mean *d*′ = 3.68 (*SD* = 1.03). All except one of these achieved a *d*′ > 1.0, most of them had even perfect performance. NH listeners performed significantly better than the CI listeners, *t*(34) = 15.59, *p* < 0.001. The dependence of the individual performance on the hearing experience is shown in Figure 6. No significant correlation of *d*′ with hearing age was found in CI listeners, *r* = 0.133, *p* > 0.05, and also not with age at testing, *r* = 122, *p* > 0.05. In contrast, a significant correlation between age and *d*′ was found in NH listeners, *r* = 0.598, *p* < 0.01. Notably, the two youngest NH listeners provided the lowest *d*′ values. The mean decision criterion *c* = -0.31 (*SD* = 0.41) was significantly different from zero, *t*(11) = -2.58, *p* < 0.05 for the group of CI listeners, indicating a bias toward judging the melodies’ ending as “good.” For the NH, the mean decision criterion was *c* = +0.03 (*SD* = 0.31) and not significantly different from zero, *t*(23) = 0.43, *p* > 0.05. Thus, NH listeners did not show any bias.

### Discussion

The function of a chord in conclusion of a harmonic phrase was tested by replacing the musically expected consonant tonic chord with a dissonant chord. This was expected to present a distinct violation of harmonic rules and thus to be far less satisfactory, at least for children with normal hearing and sufficient (implicit) musical experience. The results of the NH listeners (except those of the two youngest ones) confirmed this hypothesis. In contrast, none of the CI listeners achieved a performance above chance level. This striking difference between NH and CI listeners replicated the results in postlingually deafened adult CI listeners (Knobloch et al., 2018). However, Knobloch et al. (2018) also observed that harmonic function is not completely unaccessible when listening through a CI, since a single CI participant of their cohort was fairly successful in this task, and even more reliable in detecting tonic chords shifted by a semitone. This is corroborated by findings of an ERP component elicited by Neapolitan sixth chords (Koelsch et al., 2004). In chord progressions, this inherently consonant chord is musically quite irregular. The ERP component was regarded as a correlate of the neural representation of a violation of musical syntax. This component was found in NH listeners, but was less pronounced in CI listeners, indicating that a syntactic irregularity was registered by CI listeners to some extent. In contrast, the psychoacoustic study by Knobloch et al. (2018), as well as the present experiment, directly asked the listeners if they perceived a music-syntactic completion of the phrase. Whereas those harmonic violations do not seem to be more explicit than the ones used here and by Knobloch et al. (2018), it is unclear why they were evident for CI listeners in the ERP measurements but not registered by the listeners in the present behavioral experiment. Because the NH listeners showed a significant improvement with age, it is possible that single prelingually deaf CI listeners might develop some ability to recognize authentic cadences.

## General Discussion

**FIGURE 7:**
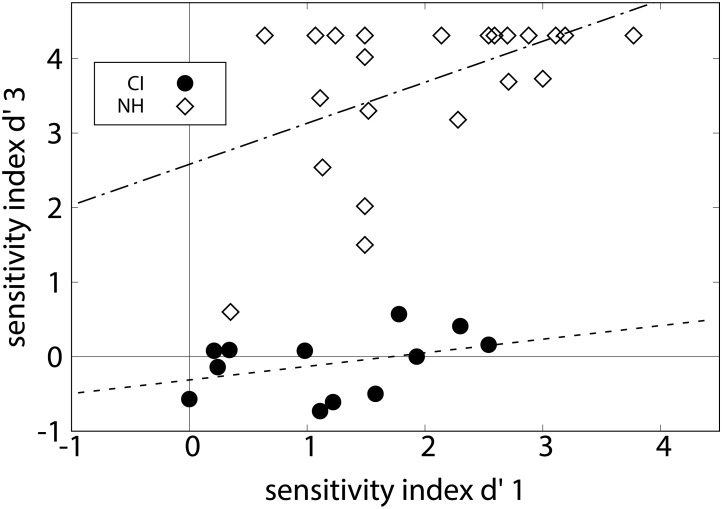
Individual sensitivity indices *d*′ of experiment 3 are plotted versus the corresponding values of experiment 1 for CI listeners (circles) and NH listeners (rhombs) with corresponding correlation lines.

The current study focused on how prelingually deafened, juvenile CI listeners perceive different musical chords in isolation (experiments 1 and 2) and within a music-syntactical context (experiment 3). NH juveniles at an age comparable with the CI experience of the CI listeners served as a control group. In order to test if individual participants excel or fail in both discrimination experiments, the correlations of the *d*′ values of the experiments were calculated (Figure 7). A significant correlation of the discrimination experiments 1 and 3 was found in the control group of NH juveniles (*r* = 0.482, *p* < 0.05) but not in the CI listeners (*r* = 0.376, *p* > 0.05). The latter result is explained by the fact that none of the CI listeners was able to successfully do the task of experiment 3. The correlation of both experiments in the NH listeners might suggest that good chord discrimination is linked with – or even a prerequisite for – registering the conclusiveness of a cadence. However, both abilities might also be governed by other factors such as age at testing or technical device limitations. No significant correlation of the slope of experiment 2 with the *d*′ values of experiments 1 and 3 was found in the NH listeners or in the CI listeners.

Discrimination of two chords can be regarded as a relatively basic capacity that does not even require a concept of consonance, and is available to 6-year-olds already (Hair, 1973). The perception of vertical consonance is also available at this age (Costa-Giomi, 2003). In contrast, reliable processing of cadences is hardly found before an age of six (Schellenberg et al., 2005). Such knowledge of harmonic syntax can thus be regarded as a higher stage of harmonic awareness (for a review, see Trainor and Hannon, 2013).

In total, the performance of the NH listeners in experiment 1 did not depend significantly on their age. Taken together with the average *d*′ of 2.0, the ability to discriminate chords is obviously developed at the age of the participants. It is nevertheless striking that most of the younger participants (with an age of six to about nine) provide lower *d*′ values here (see Figure 2). Notwithstanding the absence of a significant correlations, we thus cannot rule out that this performance might still develop further with age. The present experiments also cannot disentangle whether this is related to the implicit acquisition of harmonic concepts, or just task-related competence. Developing harmonic concepts might also explain why, in experiment 2, the slope of the chord type preferences became significantly steeper with age, suggesting that chord preference may be shaped by musical experience. Age at testing was more strongly correlated with categorization of cadences (experiment 3) than with the slope of the chord type preferences (experiment 2), suggesting a stronger developmental component for perception of harmonic syntax as compared to vertical consonance. Note that the average young NH listeners were comparably accurate as the adult NH listeners were in a similar task using abstract cadences (Knobloch et al., 2018). Taken together, our findings corroborate that harmonic concepts develop with age and are available by an age of about 8 years, and that the tests used here are appropriate for participants of that age.

The CI listeners in the present study all lacked acoustical experience in music and could, in particular, develop concepts of harmony through electric stimulation only. The effect of this experience is difficult to predict. On the one hand, electric-only listening experience might promote reliance on different features of music than with acoustic hearing, or may result in no clear concept at all. On the other hand one might argue that in cases of late, postlingual deafness, patterns and concepts developed with previous acoustic hearing may conflict with the patterns provided by electric hearing. It is, however, plausible that prelingually deaf CI listeners take longer to develop such complex harmonic competences than NH peers. In our data, chord discrimination started very low in the CI group but tended to catch up with the NH group, and the correlation with hearing age was significant. It should be noted that this increase in performance is also correlated with chronological age at testing and might therefore be due to hearing-independent development of the children. The perception of some complex, music related sounds appears to be related to hearing age rather than chronological age (DiNino and Arenberg, 2018). In the present data, these contributions cannot be separated, since hearing age and age at testing are highly correlated in the present CI participants. In experiment 2, the slopes of chord preference became also steeper with hearing age, not reaching significance. In experiment 3, no effect of hearing age was found since even the most experienced CI listener was largely unsuccessful in the task. Knobloch et al. (2018) found that only a single listener from their group of adult CI users was able to categorize abstract authentic cadences whereas the majority of participants was not. It remains open how far a focused musical training might help to improve the registration of harmonic syntax.

Beyond the hearing experience of the listeners, technical parameters of the CI devices might also influence the perception of musical stimuli, such as the degraded frequency mapping, the mismatch of place pitch, limitations in rate pitch, and dynamic range compression (Limb and Roy, 2014). General conclusions on influences of the processing strategy cannot be drawn from our results due to the relatively small number of participants using a certain strategy. Participants with disabled electrodes did not show any suspicious performance. It should be noted that in 6 of the 12 CI listeners, the minimum acoustic input frequency was ≥188 Hz (Table 1). As such, F0 for many stimuli in experiments 1–3 were below the acoustic input frequency range of the CI device. For the remaining 6 CI listeners, the minimum acoustic input frequency was 100 Hz and therefore could accommodate the F0 for the experimental stimuli. Again, no evidence for a better performance especially in the chord discrimination experiment emerged. Note that a missing fundamental of the lowest note might, as in NH listeners, still be perceived using the higher harmonics (cf. Hu and Loizou, 2010). The discriminating note in the chord pairs of experiment 1 and 2 was always one of the top two notes and thus in the audible range. Furthermore, the second lowest note was always the octave of the chord’s root. Thus, in case the root note would be inaudible, the chord was still musically of the same type.

The pitch of harmonic tone complexes depends on the regular frequency spacing of its components (Terhardt, 1998). The CI might compromise that spacing because the coded frequency often deviates by more than 50% (i.e., a musical fifth) from the best frequency of the stimulated site of the auditory nerve (Landsberger et al., 2015). Such a mismatch is likely to affect the perceived pitch relations within the musical interval, in particular for higher frequencies that stimulate the more basal electrodes. In addition, because the frequency allocation of the sound processor is fixed, components of the chord tones may fall into common bands in an uncontrolled manner. Knobloch et al. (2018) hypothesized that this might cause roughness patterns that are transmitted through temporal envelope fluctuations by the CI, and differ from those perceived with normal hearing, an effect which has not been studied systematically in CI listeners. This would modify the perceived dissonances in our experiments 2 and 3. The adult CI users from Knobloch et al. (2018) and the present juvenile CI users both judged the pleasantness of the different chord types similarly to the expected order. The influence of filter bandwidths on the perceived consonance of tone combinations would represent an interesting research question of a future study. In experiment 3, the roughness of a chord might vary with the position of the F0 of the chord root within the device’s frequency band. This effect was ruled out by choosing different keys (and thus different root F0 values) for the songs of the third experiment.

Nevertheless, the results of experiment 1 and 2 showed that when presented in isolation, chords could be discriminated by approximately half of CI participants, and that dissonance was perceived similarly by NH and CI listeners. These findings may not have been expected, since the chords of a pair differ by just a semitone in one or two of the chord tones, and the frequency discrimination of single tones, whether for pure tones or harmonic complex tones, is in the order of several semitones (see, e.g., Pretorius and Hanekom, 2008; Kang et al., 2009). Thus, the perceived difference of the chords obviously relies on more complex cues than pitch discrimination, such as temporal envelope fluctuations generated from the roughness of the chord tones. The findings by Koelsch et al. (2004) and Knobloch et al. (2018) corroborate that consonant and dissonant chords may be perceived differently even with that small contrasts.

The inability of the CI listeners to detect a correct ending in experiment 3, however, suggests that in chord sequences they fail to register the syntactical role of the cadences. Roy et al. (2014) asked children listening through a CI to discriminate chord triplets constructed from a chord x framed by two major chords I (I-x-I). These triplets might be regarded as a minimal cadence, although they were not controlled for harmonic syntax as are the present data. The CI listeners had comparable accuracy as their NH peers, thus showing surprisingly little challenge in a harmonic sequence task. Caldwell et al. (2016) asked adult CI users to rate the pleasantness of melodies accompanied with different levels of dissonance. Different from NH, CI listeners judged all accompaniments to be similarly pleasant, suggesting that they did not perceive any degree of dissonance. Due to the combination of the three experiments, the present results can provide a more detailed insight into the perception of harmonic sequences than the above studies. The results suggest that although CI listeners are capable of some degree of harmony perception (e.g., chord discrimination, chord preference), the degraded CI signal and the limited experience with harmonic syntax may have limited CI performance for more challenging perceptual tasks (e.g., perception of the tonic in an authentic cadence). It is possible that prolonged and focused training might strengthen the perception of harmony of CI listeners. A possible access might be available by comparison of acoustic and CI presentation in single-sided users with a certain musical experience.

## Summary And Conclusion

Among the fundamental challenges of listening through a CI is the accurate reception of music. Prelingually deafened, early-implanted children must develop music perception via the degraded signal provided by the CI, which may limit the beneficial impact of music on their development. Nevertheless, the present findings suggest that consonance is somewhat accessible to at least some CI users. Isolated major chords are, on average, perceived as more consonant than augmented chords, thus providing access to elements of the language of Western music. Discrimination of chords furthermore develops with listening experience. In contrast, typical music-syntactical chord sequences, such as cadences indicating an ending of a phrase, are hardly available, just as it was found in postlingually deaf CI users. It remains to be investigated, to what extent a focused, long term musical training might foster the processing of musical syntax.

## Author Contributions

VZ, MB-B, and JV contributed to the conception and design of the study and performed the data analysis. MB-B designed the musical stimuli and the experimental user interface. VZ ran the experiments. MZ helped with choice of potential listeners and provided the fitting of the CI processors of the listeners. VZ wrote the first draft of the manuscript. All authors contributed to manuscript revisions, read, and approved the submitted version.

## Conflict of Interest Statement

The authors declare that the research was conducted in the absence of any commercial or financial relationships that could be construed as a potential conflict of interest.
