# Supplementary material for: Harmony Perception in Prelingually Deaf, Juvenile Cochlear Implant Users
Source: Front Neurosci. 2019 May 8;13:466. doi: 10.3389/fnins.2019.00466 (PMC6518352; doi:10.3389/fnins.2019.00466)

Fuchs, du hast die Gans gestohlen

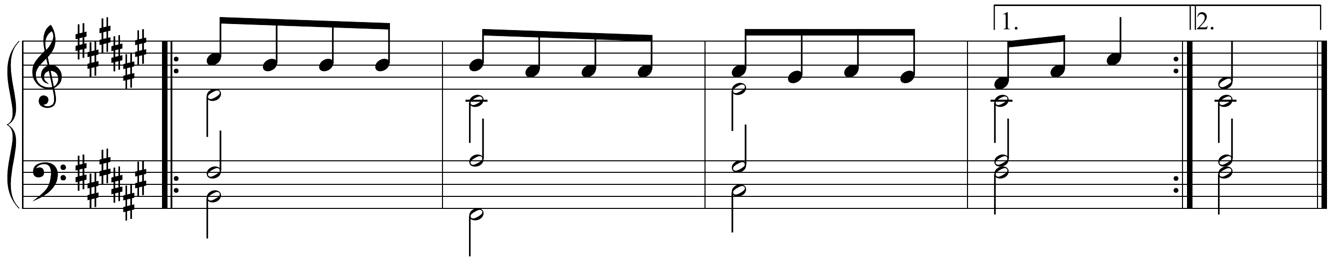

Grün sind alle meine Kleider

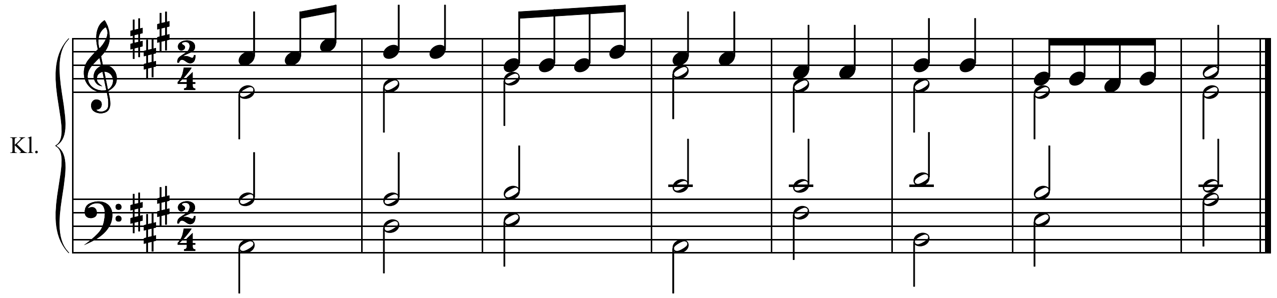

Guten Abend, gute Nacht

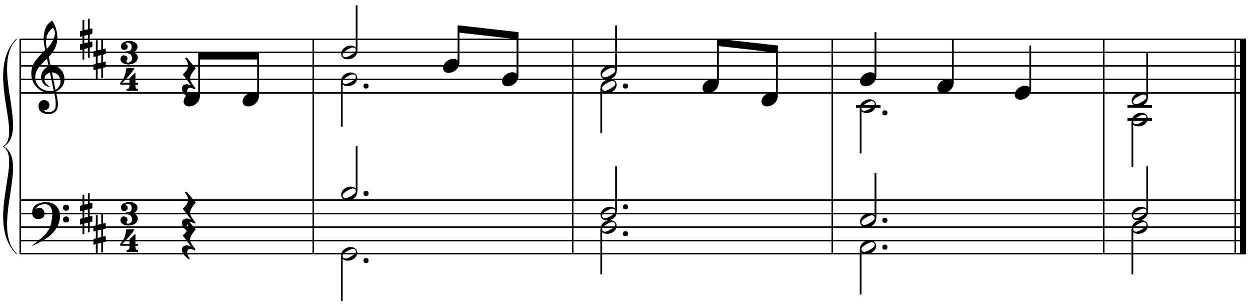

Happy Birthday

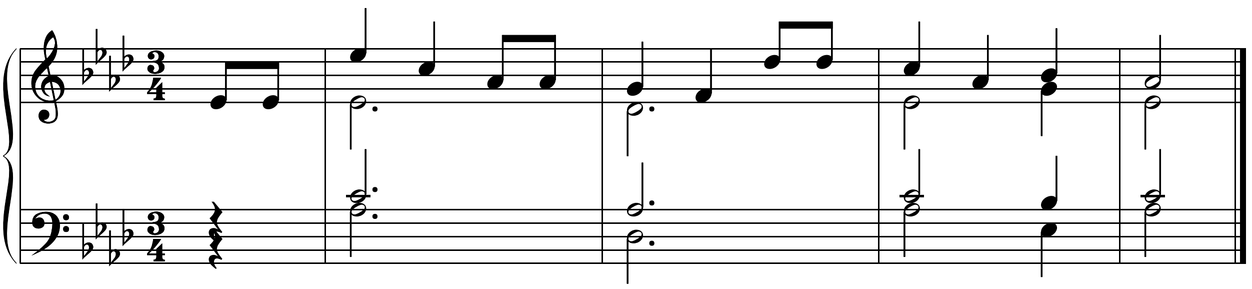

In der Weihnachtsbäckerei

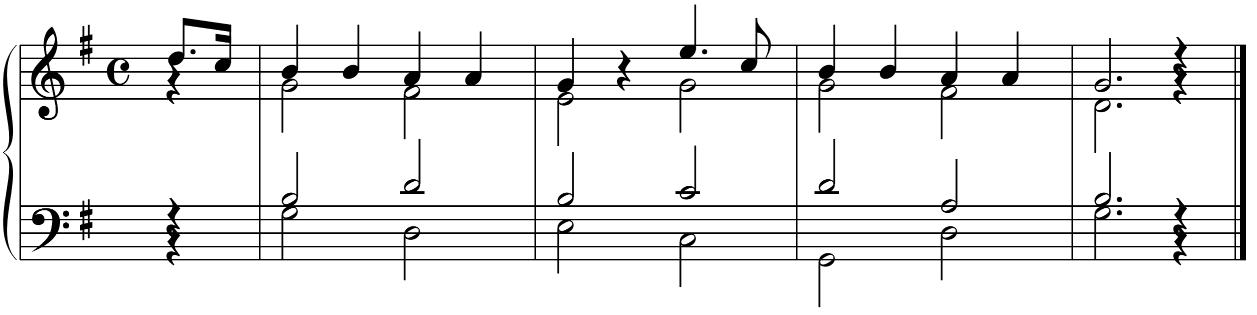

Sandmann, lieber Sandmann

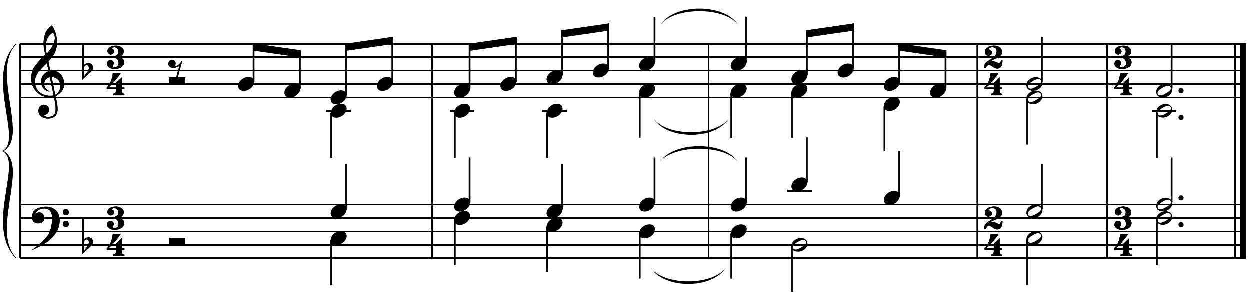

Schneeflöckchen, Weißröckchen

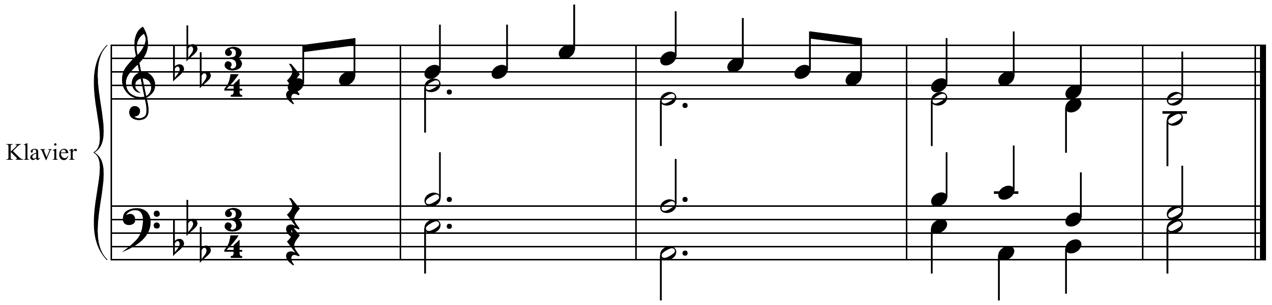

Spannenlanger Hansel

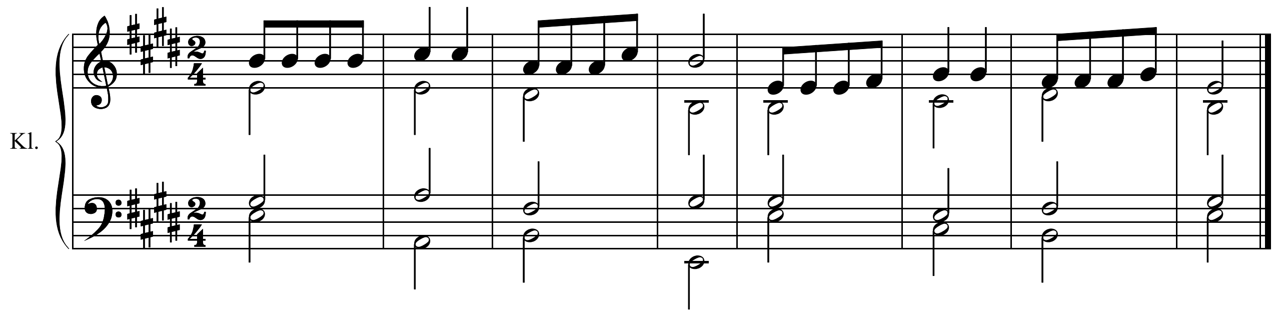

Supplement: Supplementary file 1 [file Presentation_1.pdf]
